# Sensitivity of diffusion tensor imaging to regional mixed cerebrovascular pathology

**DOI:** 10.1093/braincomms/fcaf193

**Published:** 2025-05-22

**Authors:** Jennifer K Ferris, Julia Dahlby, Shie Rinat, Brian Greeley, Joel Ramirez, Sandra E Black, Lara A Boyd

**Affiliations:** Graduate Programs in Rehabilitation Sciences, University of British Columbia, Vancouver, Canada V6T 1Z3; Gerontology Research Centre, Simon Fraser University, Vancouver, Canada V6B 5K3; Graduate Programs in Rehabilitation Sciences, University of British Columbia, Vancouver, Canada V6T 1Z3; Department of Physical Therapy, University of British Columbia, Vancouver, Canada V6T 1Z3; Graduate Programs in Rehabilitation Sciences, University of British Columbia, Vancouver, Canada V6T 1Z3; Department of Physical Therapy, University of British Columbia, Vancouver, Canada V6T 1Z3; Department of Physical Therapy, University of British Columbia, Vancouver, Canada V6T 1Z3; LC Campbell Cognitive Neurology Research Unit, Dr. Sandra Black Centre for Brain Resilience and Recovery, Hurvitz Brain Sciences Research Program, Sunnybrook Research Institute, Toronto, Canada M4N 3M5; Graduate Department of Clinical Psychological Science, University of Toronto Scarborough, Toronto, Canada M1C 1A4; LC Campbell Cognitive Neurology Research Unit, Dr. Sandra Black Centre for Brain Resilience and Recovery, Hurvitz Brain Sciences Research Program, Sunnybrook Research Institute, Toronto, Canada M4N 3M5; Department of Medicine, Division of Neurology, Sunnybrook Health Sciences Centre and University of Toronto, Toronto, Canada M4N 3M5; Graduate Programs in Rehabilitation Sciences, University of British Columbia, Vancouver, Canada V6T 1Z3; Department of Physical Therapy, University of British Columbia, Vancouver, Canada V6T 1Z3; Djavad Mowafaghian Centre for Brain Health, University of British Columbia, Vancouver, Canada V6T 1Z3

**Keywords:** white matter hyperintensities, cerebral small vessel disease, stroke, diffusion tensor imaging, lesion volume

## Abstract

Diffusion tensor imaging is a candidate biomarker in cerebrovascular disease. Yet, little is known about the sensitivity of diffusion tensor imaging to mixed forms of cerebrovascular pathology: stroke and white matter hyperintensities. We evaluated the sensitivity of diffusion tensor imaging to regional lesion load, considering both stroke and white matter hyperintensity lesions. 65 older adults and 39 individuals with chronic stroke underwent diffusion tensor imaging and comprehensive cerebrovascular lesion segmentation. We tested relationships between fractional anisotropy or mean diffusivity and cerebrovascular lesions with linear mixed effects regression. In older adults, tract microstructure related to white matter hyperintensity lesion load (fractional anisotropy: *b* = −0.003, *P* = 0.003; mean diffusivity: *b* = 0.071 × 10^−4^, *P* < 0.001). In individuals with chronic stroke, tract microstructure related to stroke lesion load (fractional anisotropy: *b* = −0.041, *P* < 0.001; mean diffusivity: *b* = 1.460 × 10^−4^, *P* < 0.001), with a significant interaction between stroke and white matter hyperintensity lesion load (fractional anisotropy: *b* = 0.019, *P* < 0.001; mean diffusivity: *b* = −0.727 × 10^−4^, *P* < 0.001). Among both groups, whole-brain normal appearing white matter microstructure did not relate to whole-brain lesion volumes. Our findings provide foundational evidence for the use and interpretation of diffusion tensor imaging as a biomarker in cerebrovascular disease.

## Teaser

Ferris *et al*. investigate the sensitivity of diffusion tensor imaging to cerebrovascular lesions. Regional tract microstructure was sensitive to white matter hyperintensity and stroke lesion load; whole brain microstructure did not relate to whole brain lesion volumes. This informs the use and interpretation of diffusion tensor imaging in cerebrovascular disease.

## Graphical Abstract

**Graphical Abstract:**
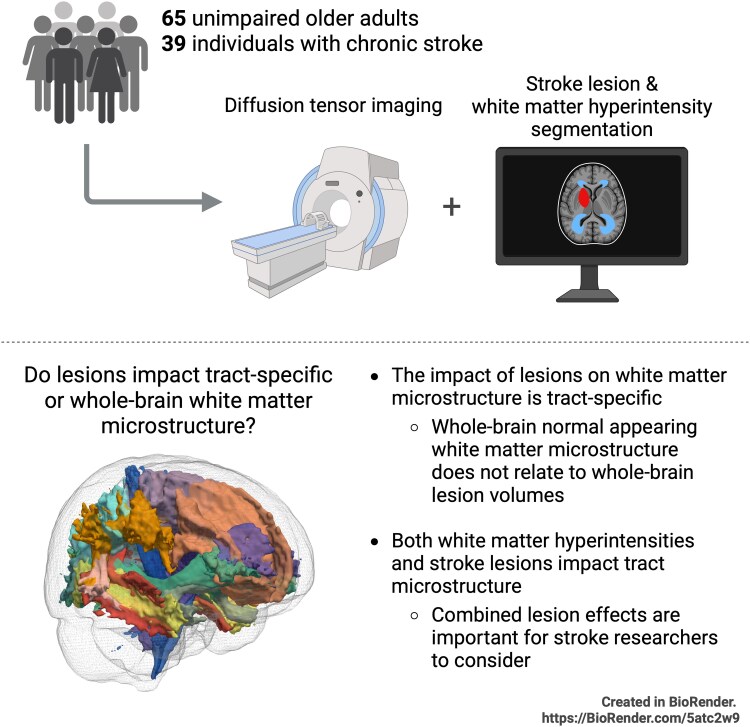

## Introduction

Diffusion tensor imaging (DTI) is a commonly used neuroimaging measure of white matter structure. DTI has emerged as a candidate biomarker for behavioural outcomes in two forms of cerebrovascular disease: stroke^1^ and white matter hyperintensities (WMHs) of presumed vascular origin.^2^ In individuals with stroke, fractional anisotropy (FA) decreases over the subacute to chronic phase of recovery^3^ and is lower in the ipsilesional hemisphere relative to the contralesional hemisphere.^4^ FA of white matter pathways has been shown to relate to post-stroke outcomes,^5^ most notably in the corticospinal tract (CST).^6^ In unimpaired older adults with WHMs, DTI metrics can sensitively discriminate between WMHs and normal appearing white matter (NAWM), with mean diffusivity (MD) being a more sensitive marker of WMHs than FA.^7-9^ DTI metrics relate to cognitive performance in individuals with WMHs,^8,10-12^ and DTI has been proposed as a potential marker of cognitive decline in aging.^2^ Altered DTI microstructure in cerebrovascular disease is interpreted as reflecting damage to white matter tracts from cerebrovascular lesions.^1,2^ However, this interpretation has yet to be fully validated as the sensitivity of DTI metrics to cerebrovascular lesion load within white matter tracts has not been directly tested. Validating the sensitivity of DTI tor regional lesion load will provide important foundational knowledge for the use of DTI as a biomarker in cerebrovascular disease.

Stroke and WMHs have largely been researched in isolation, but they are closely related. Stroke and WMHs share a common cardiometabolic aetiology,^13^ WMHs are a risk factor for stroke,^14^ and individuals who experience a stroke are more likely to have larger WMHs than age-matched older adults.^15^ Since we expect individuals with stroke to have mixed white matter damage from both stroke lesions and age-related WMHs, both lesion types may be meaningful contributors to changes in white matter DTI microstructure. However, we do not understand how sensitive DTI metrics are to the presence of combined stroke and WMH lesions in a white matter tract. Testing the sensitivity of DTI to mixed cerebrovascular pathology will improve the use of DTI as a biomarker for stroke outcomes, because we will better be able to identify the neural drivers of stroke recovery (i.e. contributions of the stroke lesion versus co-existing WMH lesions to stroke outcomes).

The aim of this study was to test the sensitivity of DTI microstructure to chronic stroke lesions, WMHs and their combined effects in a cohort of unimpaired older adults and individuals with chronic stroke. We evaluated the impact of regional lesion load on DTI microstructure across major white matter pathways in the brain and compared regional analyses to whole brain lesion volume and NAWM. We hypothesized that DTI microstructure would be sensitive to regional WMH and stroke lesion load. Characterizing the sensitivity of DTI to multiple forms of brain pathology is important to establish the biological validity of DTI as a biomarker, and to assess how DTI reflects the mixed neuropathological profiles that we expect to see in aging and cerebrovascular disease. This knowledge will help us refine neuroimaging biomarkers for the prediction of behavioural outcomes.

## Materials and methods

### Participants

Data for this study were pooled from the baseline data of two studies as a secondary analysis. Participants were older adults or individuals in the chronic phase of stroke recovery who completed multimodal neuroimaging between 2016 and 2020. Participants were considered eligible if they were between 40 and 80 years old. Individuals with stroke were eligible if they were in the chronic phase of recovery (>6 months after a clinically diagnosed stroke). Participants were ineligible if they: (i) had a history of seizure/epilepsy, head trauma, a major psychiatric diagnosis, neurodegenerative disorders or substance abuse; or (ii) reported any contraindications to MRI. All participants received the Montreal Cognitive Assessment (MoCA),^16^ administered by a trained researcher. All individuals with chronic stroke completed the upper extremity portion of the Fugl–Meyer assessment of motor impairment.^17^ Informed consent was obtained for each participant in accordance with the Declaration of Helsinki. Data collection were performed at the University of British Columbia, in Vancouver, Canada. All aspects of the study protocol were approved by the University of British Columbia research ethics boards.

### MRI analyses

See the Supplementary Materials for details on MRI acquisition and pre-processing procedures.

#### Regions of interest

**Figure 1:**
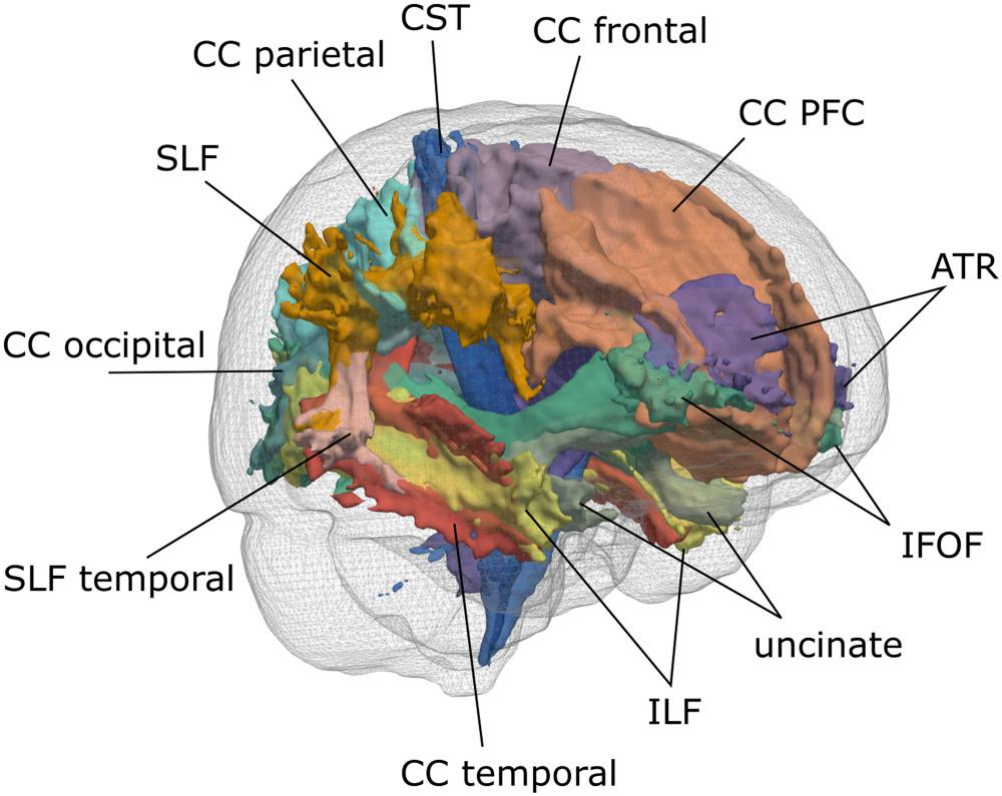
**White matter regions of interest.** Regions of interest were derived from the Johns Hopkins University white matter atlas^18^ and TCATT atlas.^19^ ATR, anterior thalamic radiations; CC, corpus callosum; IFOF, inferior fronto-occipital fasciculus; ILF, inferior longitudinal fasciculus; SLF, superior longitudinal fasciculus.

Figure 1 shows an overview of included white matter regions of interest (ROIs). ROIs were taken from the John’s Hopkins University white matter atlas^18^ and binarized with a probability threshold of 0.1.^20,21^ Corpus callosum ROIs were taken from the transcallosal tract template (TCATT) atlas.^19^ To calculate regional lesion load we calculated the weighted overlap between each stroke and WMH mask and the ROI in MNI space, according to previously published methods.^22^ Lesion load calculations were performed in the plane that captured the cross-sectional area of the tract’s trajectory (i.e. sliced in the coronal plane for anterior thalamic radiations (ATR), sliced in the axial plane for CST). Lesion load is then calculated as the sum of the overlapping cross-sectional area between the lesion and tract, adjusted by the maximum cross-sectional area of that tract. This quantifies the extent of overlap between a lesion and a white matter tract, while accounting for changing tract diameter along its length. To calculate regional DTI metrics: ROIs were moved to T1 space and eroded to subject-specific anatomy by removing voxels from the ROI containing grey matter or cerebrospinal fluid. Next, eroded ROIs were moved to DTI space, and mean FA and MD were extracted.

### Statistical analysis

Statistical analyses were performed in R (programming environment v4.0.4). Whole-brain lesion volumes and tract-specific lesion load measures were positively skewed and were log-transformed prior to statistical analysis. Predictor variables were group mean cantered and standardized. Separate models were performed with FA or MD as the outcome measures of interest and analysed separately by group (older adults and individuals with chronic stroke). Data from individuals in the older adult group were collected across two MRI scanners, thus MRI scanner was included as a factor to account for potential MR-related variance. Data from individuals in the chronic stroke group were collected on a single MRI scanner, thus MRI scanner was not included in models for the chronic stroke group. There were no missing data for included participants. Linear mixed effects models were fit with the R packages lme4^23^ and lmerTest,^24^ and the significance of predictors in the model was assessed with Satterthwaite’s approximation.^25^ The alpha threshold for significance was set at *P* < 0.05.

First, we tested whether regional lesion load related to regional DTI metrics across all major white matter tracts in the brain. Cingulate and cingulum hippocampus had low WMH and stroke lesion load across the sample (lesions present in <10% of the older adult sample and <30% of the chronic stroke sample); thus, these regions were excluded from regional analyses because of this high proportion of missing data. We constructed linear mixed effects models with regional FA and MD as outcome variables. For the older adult group, we entered the following fixed effects: age, MoCA score and regional WMH lesion load; and the following random effects: MRI scanner with a nested random effect of participant ID, cerebral hemisphere and ROI. For the chronic stroke group, we entered the following fixed effects: age, MoCA score, months since stroke and a regional WMH lesion load by regional stroke lesion load interaction; and the following random effects: participant ID, cerebral hemisphere and ROI. The random effects structure in these linear mixed effects models allowed us to combine data from each major white matter tract to test relationships between regional lesion load and regional DTI metrics, while accounting for variability between and repeated measures across scanner, participants, hemispheres and each individual ROI.

Next, we conducted a follow-up analysis to test whether whole-brain lesion volumes were related to whole-brain NAWM microstructure. In this analysis, we used whole-brain NAWM masks to extract a single mean FA and MD value for each participant. We constructed multiple linear regression models with NAWM FA or MD as the outcome measures. For the older adult group, we entered the following predictor variables: age, MoCA score, MRI scanner and whole-brain WMH volume. For the chronic stroke group, we entered the following predictor variables: age, MoCA score, months since stroke and a whole-brain WMH volume by whole-brain stroke volume interaction.

## Results

**Figure 2:**
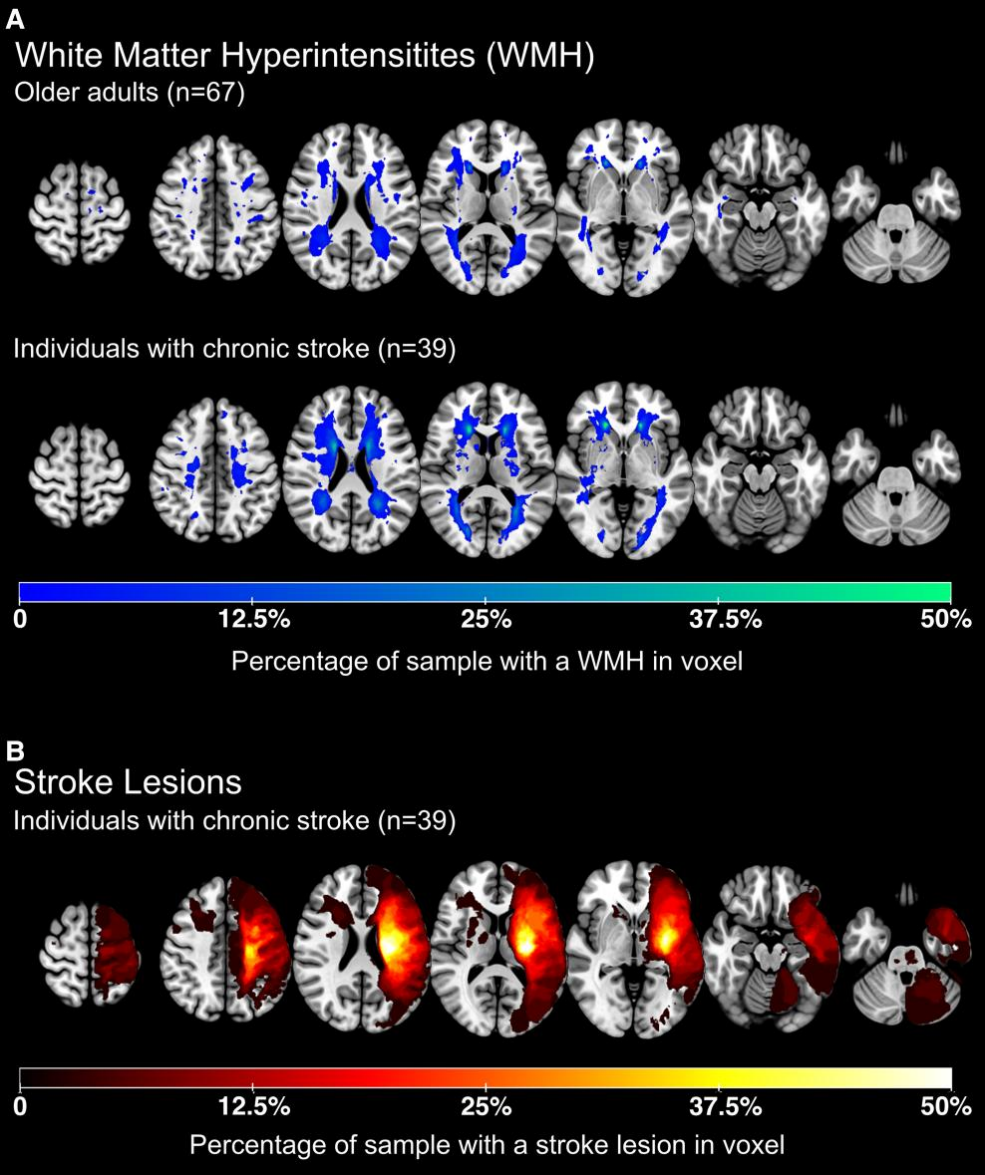
**Lesion overlap images for [(A) white matter hyperintensities (WMH) and (B) stroke lesions].** (**A**) WMHs are presented separately by group (unimpaired older adults and individuals with chronic stroke). (**B**) Stroke lesions were flipped along the L/R axis so that all symptomatic strokes (contralateral to the impaired upper extremity) were visualized in the left hemisphere (note: images are in radiological orientation).

Sixty-five unimpaired older adults (age range: 46–80) and 39 individuals with chronic stroke (age range: 45–80) were included in this study. All participants were successfully processed through MRI processing steps. Table 1 presents participant demographics. Relative to the unimpaired older adults, individuals with chronic stroke had fewer females, lower cognitive function (indexed by MoCA scores) and larger WMHs volumes. Figure 2 presents lesion overlap images for WMHs and stroke lesions across the sample. We tested for potential sex differences in imaging metrics, presented in Supplementary Table 1. There were no significant sex differences in whole-brain WMHs volumes, stroke volumes, FA or MD within either the older adult or chronic stroke groups (all *P* > 0.05; Supplementary Table 1).

**Table 1:** Participant demographics and group comparisons between unimpaired older adults and individuals with chronic stroke

	Older adult group	Chronic stroke group	*P*
*N*	65	39	
Age			
Mean (SD)	64 (8)	66 (8)	0.261
Sex			
*n* (%) females	41 (63%)	12 (31%)	**0.002** ^a^
MoCA			
Mean (SD)	27 (2)	24 (4)	**<0.001** ^b^
% ≥ 26	76%	49%	
WMH volume (mL)			
Median [IQR]	0.377 [0.137–1.071]	1.901 [1.126–5.645]	**<0.001** ^b^
Stroke volume mL			
Median [IQR]	n/a	5.763 [1.499–45.414]	
Months since stroke			
Mean (SD)	n/a	71 (61)	
Fugl–Meyer score			
Mean (SD)	n/a	44 (20)	

Bold values indicate statistical significance (*P* < 0.05).

MoCA, Montreal cognitive assessment; WMH, white matter hyperintensities; SD, standard deviation; IQR, interquartile range.

^a^Group comparison with χ^2^ test.

^b^Group comparison with independent samples *t*-test.

**Figure 3:**
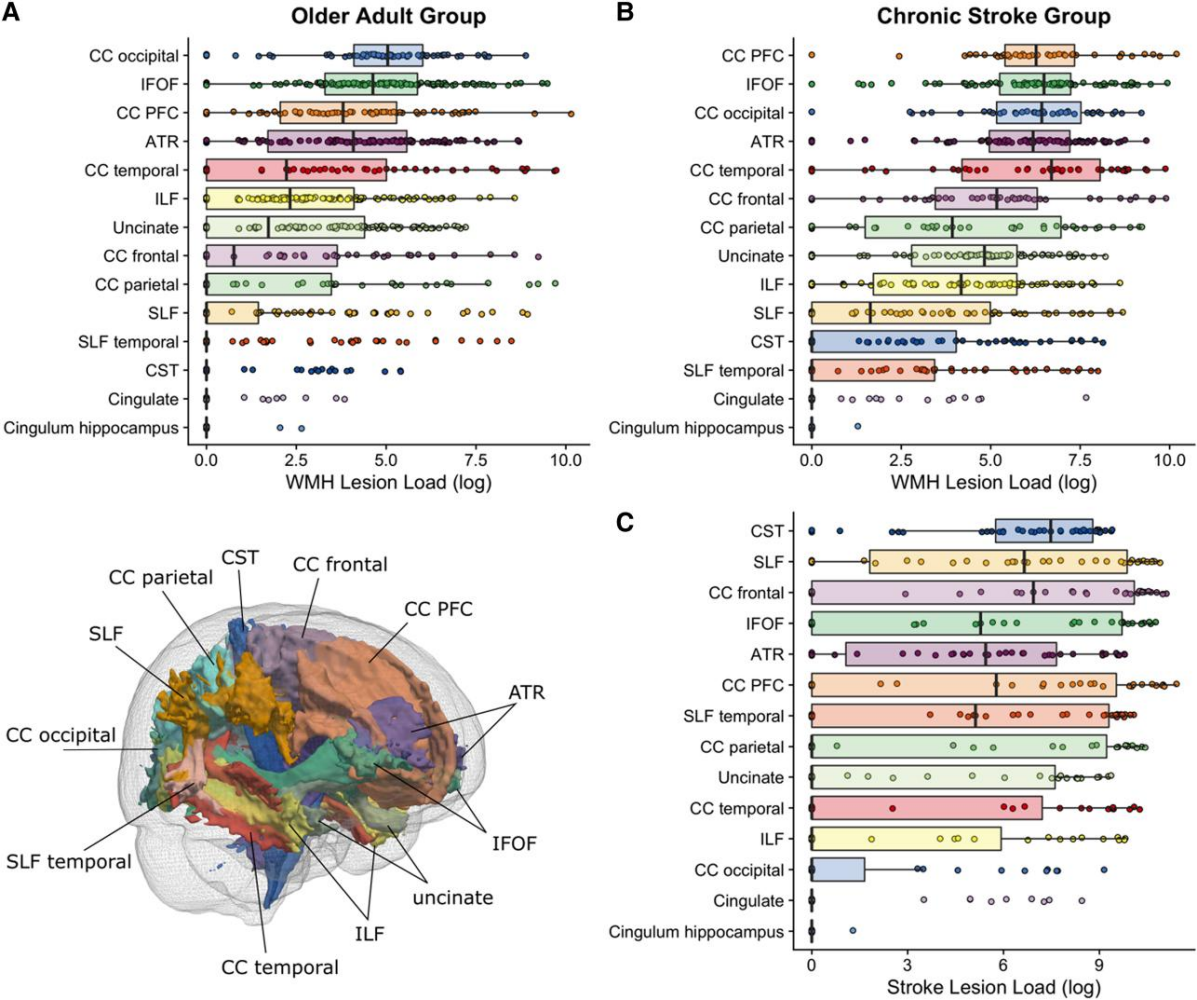
**Tract-specific lesion loads.** Lesion distributions across major white matter tracts (visualized in 3d in the inset). Lesion loads were computed as the weighted lesion load of the overlap between the lesion masks and the atlas tract. Each data point in this figure represents a participant’s lesion load in each white matter tract. (**A**) WMH lesion load in the older adult group. (**B**) WMH lesion load in the chronic stroke group (*n* = 65). (**C**) Stroke lesion load in the chronic stroke group (*n* = 39). ATR, anterior thalamic radiations; CC, corpus callosum; CST, corticospinal tract; IFOF, inferior fronto-occipital fasciculus; ILF, inferior longitudinal fasciculus; IFOF, inferior fronto-occipital fasciculus; PFC, prefrontal cortex; SLF, superior longitudinal fasciculus.

Tract-specific lesion loads are plotted in Fig. 3. We tested whether tract microstructure differed between the older adult and chronic stroke groups. FA was lower and MD higher in the chronic stroke group in every ROI used in the current analysis relative to the unimpaired older adult group (*P*’s < 0.01). Full results from group comparisons of tract microstructure are presented in Supplementary Table 2.

### Whole-tract white matter microstructure is sensitive to regional lesion load

Outcomes of linear mixed effects models are presented in Tables 2 and 3 for regional white matter tracts and whole-brain NAWM, respectively. Models were tested for multicollinearity, and all predictors were acceptable (all variance inflation factor scores <2.5). In the unimpaired older adult group, regional WMH lesion load was significantly related to regional FA and MD (Table 2). In the chronic stroke group, the main effect of regional stroke lesion load and the WMH * stroke lesion load interaction were significantly related to FA and MD, with no main effect of WMH lesion load (Table 2). We conducted a follow-up analysis to confirm these findings were region-specific effects by testing relationships between whole-brain lesion volumes and whole-brain NAWM FA and MD. Whole-brain WMH and stroke volumes did not relate to NAWM FA or MD in the older adult or chronic stroke groups (Table 3).

**Table 2:** Regional white matter tract FA and MD models

	FA		MD	
Predictor	*b*	*P*	*b*	*P*
Older adult group				
Age	**−0.007**	**0.009**	**0.148 × 10^−4^**	**<0.001**
MoCA	0.003	0.225	**−0.092 × 10^−4^**	**0.015**
WMH LL	**−0.003**	**0.003**	**0.071 × 10^−4^**	**<0.001**
Chronic stroke group				
Age	0.001	0.865	−0.079 × 10^−4^	0.617
MoCA	0.006	0.322	−0.184 × 10^−4^	0.227
Months since stroke	−0.003	0.645	−0.038 × 10^−4^	0.792
WMH lesion load	−0.003	0.347	0.048 × 10^−4^	0.647
Stroke lesion load	**−0.041**	**<0.001**	**1.460 × 10^−4^**	**<0.001**
WMH * stroke lesion load	**0.019**	**<0.001**	**−0.727 × 10^−4^**	**<0.001**

Results from linear mixed effects models testing relationships between regional lesion load (for WMHs and stroke lesions; log-transformed) and regional FA and MD across 12 major white matter tracts. Cells present standardized parameter estimates and *P*-values. Bold values indicate statistical significance (*P* < 0.05).

**Table 3:** Whole-brain normal-appearing white matter FA and MD models

	FA		MD	
Predictor	*b*	*P*	*b*	*P*
Older adult group				
Age	−0.003	0.172	0.153 × 10^−4^	**0.002**
MoCA	0.003	0.146	−0.074 × 10^−4^	0.073
Scanner	−0.007	0.113	−0.161 × 10^−4^	0.064
WMH volume	−0.005	0.053	0.014 × 10^−4^	0.763
Chronic stroke group				
Age	0.001	0.934	0.069 × 10^−4^	0.614
MoCA	0.006	0.313	−0.163 × 10^−4^	0.176
Months since stroke	−0.004	0.477	0.056 × 10^−4^	0.616
WMH volume	−0.003	0.570	0.075 × 10^−4^	0.481
Stroke volume	−0.009	0.239	0.088 × 10^−4^	0.560
WMH * stroke volume	−0.002	0.727	0.041 × 10^−4^	0.756

Results from linear regression models testing relationships between whole-brain lesion volumes (WMHs and stroke lesions; log-transformed) and normal-appearing white matter FA and MD. Cells present standardized parameter estimates and *P*-values. Bold values indicate statistical significance (*P* < 0.05).

## Discussion

Here, we examined the sensitivity of DTI microstructure (FA and MD) to regional WMHs and stroke lesions. Generally, we found that regional tract microstructure relates to regional lesion load. For unimpaired older adults, tract microstructure related to WMH lesion load. For individuals with chronic stroke, tract microstructure related to stroke lesion load and the interaction between WMH and stroke lesion load. These findings were region specific as whole-brain NAWM microstructure did not relate to whole-brain WMH or stroke lesion volumes. Our results indicate that DTI microstructure has sensitivity and specificity to regional lesion location and in individuals with chronic stroke DTI microstructure is sensitive to multiple forms of cerebrovascular pathology occurring within the same white matter tract. Together, our results reveal the sensitivity of DTI to lesion topography. These findings are important in the use and interpretation of DTI as a biomarker in cerebrovascular disease.

### Regional effects of lesion load on white matter microstructure

Our study suggests that DTI is a sensitive metric of the extent of injury to white matter pathways. There were regional lesion load effects on tract-specific microstructure across 12 major white matter pathways in the brain. In unimpaired older adults, tracts with greater WMH lesion load had lower FA and higher MD. This is congruent with previous studies in older adults showing relationships between regional WMH lesions and tract microstructure.^26,27^ In individuals with chronic stroke, stroke lesion load had the largest effect on tract microstructure, with no main effect of WMH lesion load. Stroke lesions have previously been shown to impact tract microstructure in a rodent model,^28^ but this relationship has not previously been tested in human MRI research. Our studies are congruent with past animal model research and suggest that DTI is a sensitive metric of extent of injury to human white matter pathways. By contrast, whole-brain lesion volumes (WMHs and stroke lesions) did not relate to whole-brain NAWM microstructure. This parallels findings that regional lesion location, but not whole-brain lesion volumes, relates to cognitive performance in older adults WMHs^29^ and individuals post-stroke,^30^ suggesting that DTI metrics are tract-specific as are the functional consequences of tract injury.

Importantly, we found that WMH lesion load interacted with stroke lesion load to impact tract microstructure in individuals with chronic stroke. This suggests that DTI can index cumulative lesion load in a tract from multiple intersecting forms of brain pathology. This finding is important in the clinical interpretation of relationships between tract microstructure and stroke outcomes. For example, tract microstructure in anterior regions of the corpus callosum relate to motor outcomes after stroke.^31,32^ It is possible that corpus callosum microstructure is impacted by both WMHs and stroke lesions, because anterior corpus callosum is frequently affected by WMHs (as observed in the current and previous studies^29,33,34^). Therefore, previous reports of surprising, but robust, relationships between prefrontal corpus callosum FA and motor outcomes after stroke^31,32^ might indicate an impact of WMHs on motor recovery after stroke, as has been observed in a recent study.^35^ Our data emphasize that stroke occurs over a background of concurrent WMHs and that changes to DTI microstructure can indicate impacts from stroke lesions and WMHs in combination. The influence of WMHs on stroke-related outcomes, including DTI outcomes, should be considered in future longitudinal studies of stroke recovery.

### Limitations

This analysis was cross-sectional in design, and therefore we cannot determine the time course of lesion formation and changes to tract microstructure to establish causality. However, the correlational evidence presented here strongly supports a link between lesion location and tract microstructure and provides a road map for future longitudinal studies. We used an atlas-based approach for region of interest delineation; this offers a standardized method to capture tract microstructure, but it necessarily does not account for individual subject anatomy. We balanced these trade-offs by eroding atlas ROIs to individual subject anatomy to best capture individual tract profiles. More advanced DTI models, such as neurite orientation and dispersion imaging could be applied in future research to gain deeper insights on the cellular basis for the DTI affects observed here. As this study was a secondary analysis of existing data, we did not have available data on certain relevant covariates such as vascular risk profiles. Our sample consisted of individuals with relatively low WMH burden; however, we expect our results to be generalizable to individuals with higher WMH burden as the relationships between lesion load and DTI markers would likely be stronger in this population. Our study was likely underpowered to detect sex-differences in lesion volumes or DTI microstructure. Previous research has found sex differences in WMH volumes and stroke lesion characteristics^36,37^; sex-specific differences in lesion characteristics are an important avenue for future research.

## Conclusions

The current study investigated the two most prominent lesions associated with cerebrovascular disease: stroke and WMHs. We found that white mater tract microstructure is sensitive to lesion location. In older adults, increased regional WMH lesion load is associated with decreased FA and increased MD in whole white matter pathways. In individuals with chronic stroke, both stroke lesions and WMHs impact whole-tract microstructure, which is an important consideration for future DTI work in this population. This study provides foundational knowledge for the use and interpretation of altered DTI tract microstructure in cerebrovascular disease.

## Supplementary Material

fcaf193_Supplementary_Data

## Data Availability

Data for these analyses is available upon reasonable request to the corresponding author.
