# Supplementary material for: Sensitivity of diffusion tensor imaging to regional mixed cerebrovascular pathology
Source: Brain Commun. 2025 May 22;7(3):fcaf193. doi: 10.1093/braincomms/fcaf193 (PMC12120440; doi:10.1093/braincomms/fcaf193)
Supplement: fcaf193_Supplementary_Data [file fcaf193_supplementary_data.docx]

Sensitivity of diffusion tensor imaging to regional mixed cerebrovascular pathology: Supplementary Materials

# Supplementary Methods

## MRI acquisition

MRI scans were acquired at the University of British Columbia MRI Research Centre on 3.0T Phillips Achieva or Elition scanners (Philips Healthcare, Best, The Netherlands), with parallel imaging and an eight-channel and thirty-two-channel sensitivity encoding head coil, respectively. We acquired the following structural scans: a 3D magnetization-prepared rapid gradient-echo (MPRAGE) T1 anatomical scan, a fluid attenuated inversion recovery (FLAIR) scan, and a combined T2-weighted (T2) and proton density (PD) scan. For DTI data, a high-angular resolution diffusion imaging (HARDI) scan was acquired across 60 non-collinear diffusion gradients.

## MRI preprocessing

Structural segmentation was performed with the ﻿Semi-Automated Brain Region Extraction (SABRE) Lesion Explorer pipeline^1,2^. Briefly, T1, FLAIR, T2 and PD scans were linearly co-registered and supratentorial cerebral tissue was segmented into cerebrospinal fluid (CSF), grey matter, normal-appearing white matter, and WMHs. Stroke lesions were manually traced over co-registered T1 and FLAIR scans by a single experienced researcher (by J.K.F).

Image processing was performed with tools from the FMRIB Software Library (FSL). We used the FSL’s Brain Extraction Tool (BET)^3^ to skull-strip T1 scans. For individuals with stroke with large cortical lesions, BET often fails to identify the boundaries of the stroke lesion. To improve BET segmentation for individuals with large cortical lesions, stroke lesion masks were set to a voxel intensity roughly corresponding to grey matter and used to mask the original T1 image. BET was then performed on the masked T1 image, and the resulting binarized BET mask was used to mask the unaltered T1 image, which allows BET to follow the boundaries of the stroke lesion. BET skull strips were visually checked by a single researcher (J.K.F.). T1 scans were non-linearly registered to MNI space using FSL’s FNIRT^4^. To minimize warping in non-linear registration introduced by stroke lesions, stroke lesion masks were flipped across the sagittal midline and a copy of contralesional tissue was created, which was then used to fill in the stroke region on the T1 scan prior to non-linear registration^5^. WMH masks were incorporated in the MNI registration using a cost-function mask, and registration was performed with a warp resolution of 5mm. The quality of MNI registrations was visually confirmed by a single rater (J.K.F.).

Diffusion images were preprocessed with FSL’s diffusion toolbox (FDT)^6^. Briefly, DTI data were corrected for motion and eddy-current distortion, and the unweighted DTI volume was skull-stripped with BET. FA and MD maps were generated using DTIFIT. T1 scans were registered to the unweighted DTI volume using FSL’s FLIRT^7^ by a rigid-body linear registration with a correlation ratio cost function. Registration quality was visually checked by a single rater (J.K.F.) and manually adjusted where necessary with tkregister from Freesurfer v.6.0.

|  | Older Adults | | |  | Individuals with Chronic Stroke | | |
| --- | --- | --- | --- | --- | --- | --- | --- |
|  | Females (n = 41) | Males (n = 24) | *p* |  | Females (n = 12) | Males (n = 27) | *p* |
| WMH mL median [IQR] | 0.427  [0.174 – 1.213] | 0.256 [0.094 – 0.786] | 0.324 |  | 1.790 [1.340 – 5.348] | 2.033 [1.340 – 5.348] | 0.949 |
| Stroke mL median [IQR] | - | - | - |  | 5.314 [2.394 – 15.739] | 6.185 [0.980 – 57.496] | 0.348 |
| NAWM FA mean(SD) | 0.35 (0.02) | 0.34 (0.02) | 0.590 |  | 0.31 (0.04) | 0.32 (0.03) | 0.664 |
| NAWM MD mean (SD) | 8.18 (0.37) x 10^-4^ | 8.13 (0.36) x 10^-4^ | 0.232 |  | 8.86 (0.81) x 10^-4^ | 8.81 (0.44) x 10^-4^ | 0.780 |

Supplementary Table 1: Sex differences in imaging metrics for older adults and individuals with chronic stroke, compared with independent samples t-tests. FA: fractional anisotropy; MD: mean diffusivity; NAWM: normal-appearing white matter; WMH: white matter hyperintensity

| Region |  | **FA** | | |  | **MD** | | |
| --- | --- | --- | --- | --- | --- | --- | --- | --- |
|  |  | Older Adult | Stroke | *p* |  | Older Adult | Stroke | *p* |
| NAWM |  | **0.34 (0.02)** | **0.32 (0.03)** | **<0.001** |  | **0.82 (0.04) x 10^-3^** | **0.88 (0.06) x 10^-3^** | **<0.001** |
| ATR |  | **0.37 (0.03)** | **0.33 (0.05)** | **<0.001** |  | **0.83 (0.05) x 10^-3^** | **0.99 (0.24) x 10^-3^** | **<0.001** |
| CC frontal |  | **0.44 (0.02)** | **0.39 (0.06)** | **<0.001** |  | **0.80 (0.03) x 10^-3^** | **0.95 (0.16) x 10^-3^** | **<0.001** |
| CC occipital |  | **0.50 (0.04)** | **0.46 (0.05)** | **<0.001** |  | **0.82 (0.05) x 10^-3^** | **0.87 (0.07) x 10^-3^** | **<0.001** |
| CC parietal |  | **0.47 (0.03)** | **0.43 (0.06)** | **<0.001** |  | **0.81 (0.04) x 10^-3^** | **0.95 (0.15) x 10^-3^** | **<0.001** |
| CC PFC |  | **0.38 (0.02)** | **0.33 (0.04)** | **<0.001** |  | **0.86 (0.05) x 10^-3^** | **0.99 (0.14) x 10^-3^** | **<0.001** |
| CC temporal |  | **0.50 (0.03)** | **0.46 (0.05)** | **<0.001** |  | **0.84 (0.05) x 10^-3^** | **0.95 (0.14) x 10^-3^** | **<0.001** |
| CST |  | **0.51 (0.03)** | **0.45 (0.12)** | **<0.001** |  | **0.75 (0.03) x 10^-3^** | **0.96 (0.34) x 10^-3^** | **<0.001** |
| IFOF |  | **0.42 (0.03)** | **0.37 (0.06)** | **<0.001** |  | **0.84 (0.05) x 10^-3^** | **0.98 (0.22) x 10^-3^** | **<0.001** |
| ILF |  | **0.42 (0.03)** | **0.38 (0.06)** | **<0.001** |  | **0.82 (0.04) x 10^-3^** | **0.92 (0.23) x 10^-3^** | **<0.001** |
| SLF |  | **0.38 (0.03)** | **0.33 (0.09)** | **<0.001** |  | **0.79 (0.04) x 10^-3^** | **1.00 (0.41) x 10^-3^** | **<0.001** |
| SLF temporal |  | **0.41 (0.03)** | **0.37 (0.09)** | **<0.001** |  | **0.78 (0.04) x 10^-3^** | **0.96 (0.37) x 10^-3^** | **<0.001** |
| uncinate |  | **0.38 (0.03)** | **0.31 (0.07)** | **<0.001** |  | **0.87 (0.05) x 10^-3^** | **1.09 (0.38) x 10^-3^** | **<0.001** |

Supplementary Table 2: Group differences in tract microstructure between older adults and individuals with chronic stroke, compared with independent samples t-tests. Data presented are mean (SD). ATR: anterior thalamic radiations; CC: corpus callosum; CST: corticospinal tract; FA: fractional anisotropy; IFOF: inferior fronto-occipital fasciculus; ILF: inferior longitudinal fasciculus; MD: mean diffusivity; NAWM: normal-appearing white matter; SLF: superior longitudinal fasciculus.

References

1. Dade LA, Gao FQ, Kovacevic N, et al. Semiautomatic brain region extraction: A method of parcellating brain regions from structural magnetic resonance images. *Neuroimage*. 2004;22(4):1492-1502. doi:10.1016/j.neuroimage.2004.03.023

2. Ramirez J, Gibson E, Quddus A, et al. Lesion Explorer: A comprehensive segmentation and parcellation package to obtain regional volumetrics for subcortical hyperintensities and intracranial tissue. *Neuroimage*. 2011;54(2):963-973. doi:10.1016/j.neuroimage.2010.09.013

3. Smith SM. Fast robust automated brain extraction. *Hum Brain Mapp*. 2002;17(3):143-155. doi:10.1002/hbm.10062

4. Andersson JLR, Jenkinson M, Smith S. *Non-Linear Registration Aka Spatial Normalisation; FMRIB Technical Report TR07JA2*.; 2007. http://fmrib.medsci.ox.ac.uk/analysis/techrep/tr07ja2/tr07ja2.pdf.

5. Nachev P, Coulthard E, Jäger HR, Kennard C, Husain M. Enantiomorphic normalization of focally lesioned brains. *Neuroimage*. 2008;39(3):1215-1226. doi:10.1016/j.neuroimage.2007.10.002

6. Smith SM, Jenkinson M, Woolrich MW, et al. Advances in functional and structural MR image analysis and implementation as FSL. *Neuroimage*. 2004;23(SUPPL. 1):208-219. doi:10.1016/j.neuroimage.2004.07.051

7. Jenkinson M, Bannister P, Brady M, Smith S. Improved Optimization for the Robust and Accurate Linear Registration and Motion Correction of Brain Images. *Neuroimage*. 2002;17:825-841. doi:10.1006/nimg.2002.1132
